# Functional Properties of SAP-Based Humidity Control Plasters

**DOI:** 10.3390/polym13142279

**Published:** 2021-07-12

**Authors:** Jan Fořt, Magdaléna Doleželová, Václav Kočí, Robert Černý

**Affiliations:** Department of Materials Engineering and Chemistry, Faculty of Civil Engineering, Czech Technical University in Prague, Thákurova 7, 16629 Prague, Czech Republic; magdalena.dolezelova@fsv.cvut.cz (M.D.); vaclav.koci@fsv.cvut.cz (V.K.); cernyr@fsv.cvut.cz (R.Č.)

**Keywords:** plaster, superabsorbent polymer, porosity, moisture transport and storage properties, thermal properties, passive humidity control

## Abstract

The application of materials with high moisture storage capacity close to the interior surface presents a prospective passive method for improving indoor relative humidity conditions. In this paper, lime-cement plasters containing three different types of superabsorbent polymers (SAPs) in varying dosages are introduced and their mechanical, hygric, and thermal characteristics are analyzed in a relation to microstructure. The experimental results show a significant effect of both SAP amount and chemical composition on all functional properties of studied plasters. The incorporation of 1.5% of SAP may induce up to 2.5 better moisture buffering, thus significantly improving the passive humidity control capability. Considering overall functional parameters of SAP-modified plasters, the dosage of 1 wt.% can thus be viewed as a rational compromise between the moisture storage capability and mechanical properties. The obtained wide sets of parameters can be utilized directly as input data of computational models suitable for the assessment of the interior microclimate of residential and administrative buildings.

## 1. Introduction

The substantially outdated building stock in most parts of Europe during the last few decades enforced new laws related to energy savings and sustainability measures in the construction industry [1]. Such intentions complied with the increased efforts of scientists towards improvements in the thermal performance of building envelopes. In this sense, many research studies aimed at the investigation of thermal insulation walls, heat transfer in porous building materials, application of different material layers including insulation materials, or utilization of various admixtures including phase change materials were carried out on this account [2,3,4,5,6,7,8,9,10,11,12,13,14,15].

However, optimal energy consumption is not the only concern of advanced design methods applied in the building sector, and other factors should be taken into account [16,17,18,19]. Besides the thermal performance of building envelopes, maintenance of optimal indoor air quality without excessive or insufficient levels of humidity has to be considered as well [20,21,22]. The previous research studies showed that an optimal level between 40% and 60% relative humidity (RH) is needed to ensure the well-being and health of building occupants [23,24]. While the relative humidity above 60% provides good conditions for the proliferation of viruses and mold spores, an RH level lower than 40% can result in dry skin, eye irritation, and respiratory problems [25]. However, in the last decade, this issue was pursued only rarely.

A partial solution to the problem of maintaining appropriate relative humidity conditions in the interior can be seen in the application of materials with high moisture storage capacity close to the interior surface [26]. Highly porous materials were considered as potentially suitable admixtures to plasters and mortars by various researchers in order to access passive solutions for the abovementioned issue [27,28]. In particular, the lightweight aggregates, such as perlite, charcoal, and vermiculite, became popular for the attempts to provide the required material performance [6,29,30,31]. Notwithstanding, the effectiveness of these admixtures did not provide satisfactory results and only minor improvements were achieved. 

The application of superabsorbent polymers (SAPs) as humidity control materials in plaster mixtures was investigated only rarely in the past. Mostly, these materials were utilized as internal curing agents for the mitigation of autogenous shrinkage and crack reduction in a concrete mixture to date [32,33,34,35]. Research studies aimed at the comparison of moisture buffering potential of SAPs with porogene additives [32], phase change materials (PCMs) [36], or lightweight aggregates [37,38] presented very few exceptions in that respect. A more detailed characterization of mortars containing SAPs in relation to the indoor humidity control was reported by Senff et al. [39,40], together with basic design guidelines. However, the authors analyzed only one SAP admixture and the applied low SAP dosage resulted in a limited effect on the material properties. Given the importance of indoor air quality maintenance, as well as the insufficient description of a number of effects such as the relationship between particle size, absorption capacity, and the resulting functional properties, further research is required.

The investigations presented in this paper can be considered as a further step towards filling the gaps in the current knowledge on the effect of SAP admixtures on the properties of interior plasters since detailed datasets of material parameters of plasters modified by SAPs are missing. Contrary to the previous studies published by other researchers, three different types of SAP are subjected to an in-depth study taking into account the different particle sizes and chemical composition. In addition, a more complex view of the material properties of the designed SAP containing plasters is provided. The complete sets of basic physical characteristics, mechanical, hygric, and thermal properties can be utilized directly as input data for the computational models of heat, moisture, and momentum transport. The analysis of the links between microstructure and material properties allows one to find a rational compromise between the desired higher amount of SAP admixtures and satisfactory mechanical performance of the plasters. Finally, the methods for the assessment of passive moisture moderation capability of interior plasters are analyzed and the most effective tools for the classification of their moisture buffering properties in building envelopes are identified.

## 2. Studied Materials

Three SAPs with different chemical compositions and different mean particle diameters were used as plaster admixtures suitable for the enhancement of their passive indoor moisture control:

- Creasorb SIS—bulk density 600 kg/m^3^, d_50_ = 284 µm.
- Cabloc CT—bulk density 690 kg/m^3^, d_50_ = 61 µm.
- Hydropam—bulk density 700 kg/m^3^, d_50_ = 420 µm.

Cabloc CT is formed by cross-linked sodium polyacrylate, Creasorb SIS by cross-linked acrylamide/acrylic acid potassium salt, and Hydropam by acrylamide/acrylic acid copolymer and sodium salt. The particular materials differ in solubility as well. While Cabloc CT and Creasorb SIS do not dissolve in water, Hydropam is more viscous in contact with water, thus increases the solution viscosity compared to the other two SAPs. 

The microstructure of the studied SAPs was analyzed by scanning electron microscopy (SEM), using a Phenom XL device (Thermo Fisher Scientific Inc., Waltham, MA, USA). The vacuum-dried SAP particles were mounted on aluminum stubs using double-sided conductive carbon tape and sputter-coated with gold/palladium using a Quorum SC7620 sputter coater (Quorum Technologies Ltd., Lewes, UK). The particle size distribution of SAPs was determined by a laser particle size analyzer, Bettersizer S3 Plus (Bettersize Instruments Ltd., Liaoning, China), working on the laser diffraction principle with a measuring range from 0.01 µm to 3500 µm.

**Figure 1:**
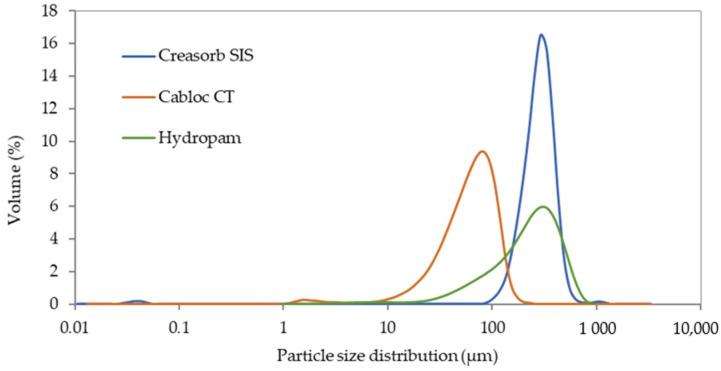
Particle size distribution of studied SAPs.

**Figure 2:**
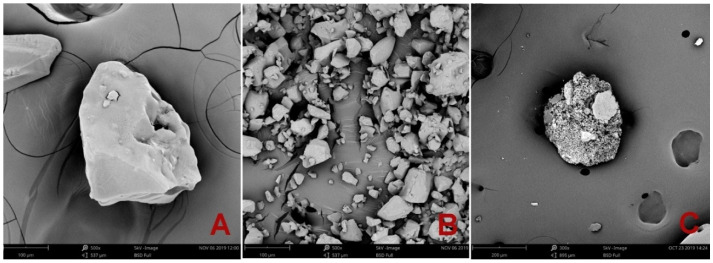
SEM images of (**A**)—Creasorb SIS; (**B**)—Cabloc CT; (**C**)—Hydropam.

The particle size distribution curves of all tested SAPs are plotted in Figure 1. The sharp peak of Creasorb SIS refers to its very uniform particles. The particle size range of Hydropam is, on the contrary, very wide. The differences between the studied SAPs are illustrated in Figure 2 where the SEM images show a significant heterogeneity. Besides the differences in particle size distribution, Hydropam SAP also exhibited a rougher surface than the other two materials.

The absorption capability in distilled and tap water by the modified particular SAPs was reported in detail in Fořt et al. [41], obtained from the modified filtration experiment. The summarized results given in Table 1 also provide information about absorption capacity of particular SAPs in cement solution w/c = 5. As one can see, Cabloc CT exhibited the highest level of absorption, while Creasorb and Hydropam reached similar results in all tested solutions. Considering the effect of dissolute cement, more distinct retardation of absorption capacity was noted for Cabloc.

As a reference material, plaster composed of lime, cement, and sand in the weight proportion of 1:1:5 was used. Within this research, Portland cement 42.5R, hydrated lime CL90, and sand <1.2 mm.

Each SAP type was applied in three different dosages ranging from 0.5 to 1.5 wt.%. The detailed composition of studied mixtures is shown in Table 2. Here, prepared plasters are denoted as follows: reference plaster—PR, plasters with Creasorb SIS—PCRE, Cabloc CT—PCAB, and Hydropam—PH, respectively. The numbers in plaster labeling correspond to the SAP dosage. Due to the increased water absorption capability of SAPs, the water dosage defined by the w/ds (water/dry substances) ratio corresponded with the constant spread diameter of 220 mm, as determined by the flow table test. While the application of Cabloc and Creasorb resulted in clumping of SAP particles and substantial worsening of workability, the addition of Hydropam caused slower sorption and formation of a viscous mix. Apparently, the incorporation of Hydropam influenced the rheology of fresh plaster mixtures to a lesser extent than Creasorb SIS and Cabloc CT. The observed findings pointed to the effect of different molecular structures of the applied SAPs on the workability of fresh mixtures as described by Liu et al. [42]. 

The samples were cast into the molds, demolded after 2 days, stored for 28 days in a highly humid environment (~75% RH), and then subjected to experimental procedures described in the following section.

## 3. Experimental Methods

### 3.1. Basic Physical Characteristics and Microstructure

At the measurement of bulk and matrix density of studied plasters, five specimens were used for each sample set. Samples were first dried out at 80 °C until constant mass and their dimensions were measured by a digital caliper to obtain the sample volume. For the matrix density determination, a helium pycnometer, Pycnomatic ATC EVO (Thermo Scientific, Waltham, MA, USA) was used. The total open porosity was calculated based on knowledge of the bulk and matrix density. 

For the characterization of the porous space of the analyzed plasters, the mercury intrusion porosimetry devices Pascal 140 and Pascal 440 (Thermo Scientific) were employed.

### 3.2. Mechanical Properties

Compressive and flexural strengths were determined according to the standard ČSN EN 1015-11 after 28 days of curing in a highly humid environment. Flexural strength was measured by a three-point bending test on three specimens with the dimensions of 40 mm × 40 mm × 160 mm. Consequently, the compressive strength was measured on the leftover specimens after the flexural strength test. 

### 3.3. Moisture Transport Properties

The water absorption coefficient was determined on five cubic samples with the 50 mm edge, which were insulated on lateral sides by epoxy resin to simulate 1D water transport. The bottom face of the sample was immersed 1–2 mm under the water level, which was kept constant by a Mariotte’s bottle with two capillary tubes. The changes in sample mass were continuously monitored by an automatic balance, the water absorption coefficient A (kg·m^−2^·s^−1/2^) was determined according to Carmeliet et al. [43], and the apparent moisture diffusivity κ (m^2^/s) was calculated using the method suggested by Kumaran et al. [44]. 

Water vapor transport properties were determined by the dry cup method. The circular samples with a diameter of 100 mm and a thickness of 30 mm were insulated on lateral sides by epoxy resin and settled into a metal cup containing silica gel below the sample. The cups were placed into a climatic chamber maintaining constant levels of relative humidity (50%) and temperature (21 °C). The samples were weighed periodically to track the mass increase. Consequently, the water vapor diffusion coefficient *D* (m^2^/s) and water vapor diffusion resistance factor *μ* (-) were calculated. 

### 3.4. Moisture Storage Properties

The sorption isotherms were measured by a dynamic method, using a DVS Advantage device (Surface Measurement Systems) to perform a modified water vapor storage test (EN ISO 9346). The moisture retention curves were determined using the cumulative pore volume curve *V*(*r*) measured by MIP. The *V*(*r*) function is decreasing, *V*(*r_min_*) = *V*_tot,_ where *V*_tot_ is the total pore volume (cm^3^/g), *V*(*r_max_*) = 0. One point of the moisture retention curve *w* = *w*(*p*_c_) can then be calculated as: *w*(*r*) = [*V*_tot_ − V(r)] *ρ*_s_, where *ρ*_s_ is the bulk density of the material, *p*_c_(*r*) = 2*σ*/*r,* where *σ* is the surface tension of water.

The moisture buffer value (MBV) was obtained using the Nordtest method [45]. The weight variations of samples exposed to 33% RH for 16 h and 75% RH for 8 h were continuously recorded at constant temperature and used for the calculation of MBV.

### 3.5. Thermal Properties

The thermal conductivity and specific heat capacity of studied plasters were determined using a surface probe of an Isomet 2114 device working on the pulse principle. The measurement was carried out on five cubic samples with a 70 mm edge in the dependence on moisture content from a dry state to water saturation.

## 4. Results and Discussion

### 4.1. Basic Physical Characteristics and Microstructure 

The basic physical characteristics are given in Table 3. The application of higher dosages of SAPs resulted in a significant decrease in the bulk density (up to 20%) and the total open porosity grew up in a corresponding way, which was due to the low density of the used SAPs ranging from 600 to 700 kg/m^3^ and varying rheology of fresh mixtures. On the other hand, the differences in the matrix density were much lower (up to 5%). The application of PCRE and PCAB resulted in more distinct changes in bulk density and porosity of plasters than in the case of PH. 

**Figure 3:**
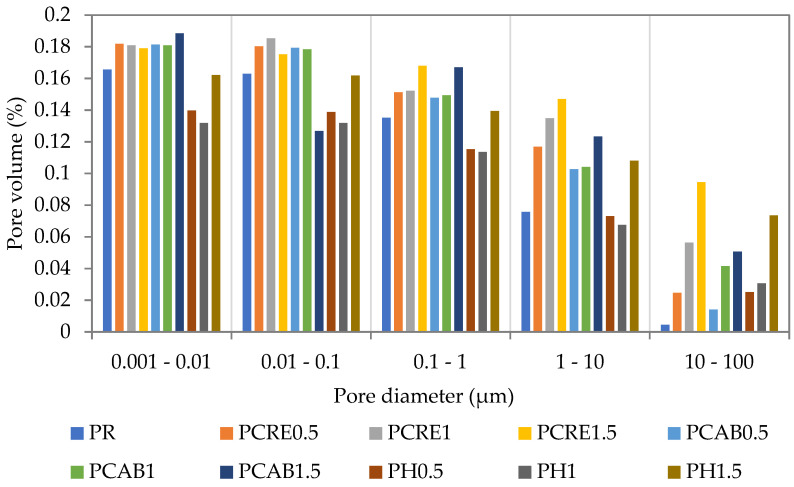
MIP of studied plasters with SAP admixtures.

The effect of applied SAPs on the pore space characteristics is shown in Figure 3. For the plasters modified by Creasorb SIS and Cabloc CT, an increased pore volume was observed in all pore size intervals. The differences were more distinct in the range of bigger pores and increased with the increasing SAP dosage. The utilization of Hydropam led to lower changes in pore volume. In the range of pores smaller than 10 μm, even a certain reduction of pore volume was found, except for the PH1.5 mixture. The different textures of PH0.5 and PH1 plasters can be attributed to internal curing induced by the better solubility of Hydropam in water. 

The results achieved in this paper for lime-cement plasters were in qualitative agreement with [46], which reported that the average pore diameter of SAP modified concrete mixtures was generally higher compared to the reference mix due to a consequent formation of a large number of pores in meso- and macroporous range. This finding complies with studies presented by other investigators [32,39], who described the effect of SAP incorporation on a pore volume increase as a result of the rapid swelling of SAP particles. The decrease in pore volume was observed only rarely [36]. SAP admixture has quite a different effect compared to other widely used admixtures due to time-dependent free water availability in the fresh mixture and consequent intense surface friction between particles. However, this effect varies substantially for particular SAP types, including the requirement on extra water dosage.

As discussed in the research paper of Tan et al. [47], the total pore volume in cement pastes (data about plaster are not available) is usually higher compared to paste without SAP admixture as a result of formed voids. The increase in porosity can also be partially attributed to water release under osmotic pressure in the form of free water and the formation of capillary pores [48].

### 4.2. Mechanical Properties

The comparison of compressive and flexural strengths after 28 days of curing is given in Table 4. As one can see, the PCRE mixtures exhibited a gradual decrease in both strength parameters, in line with the SAP dosage. A similar performance was also shown by the PCAB mixtures, except for PCAB1.5, where the drop of compressive strength was not as significant as in the case of PCRE1.5. Contrary to the PCRE and PCAB plasters, the compressive and flexural strengths of PH mixtures were improved for up to 1 wt.% SAP dosage, as compared with the reference plaster, and even 1.5 wt.% dosages resulted in only 17% compressive strength reduction. The observed enhancement of mechanical properties could be attributed to internal curing and prolongation of the hydration period, which was discussed earlier by researchers dealing with self-healing concrete design [49,50]. Considering the available knowledge from published papers, the rapid swelling of SAPs represents a major drawback for SAPs in the wider application. In other words, the substantial increase in water dosage, consequent voids formation due to SAP swelling, and adverse effect of SAPs on the rheology properties are viewed as major factors that contribute to the strength loss. Obtained findings are in agreement with the findings of Agostinho et al. [51], who have found 0.3–1 wt.% as a threshold for the preservation of rheological properties. However, dosage strongly corresponds with the type of used SAP. The obtained results are also in good agreement with the MIP measurements (Figure 3 and confirmed that both the type and dosage of SAPs are crucial parameters affecting the functional performance of lime-cement plasters. 

Summarizing the results of compressive- and flexural strength measurements, 1 wt.% dosage could be considered as a threshold value still guaranteeing a satisfactory mechanical performance of the analyzed plasters for all three applied SAPs, despite their differences in chemical composition and solubility in water. Therefore, research efforts aimed at SAP coating to decrease initial rapid swelling may lead to an improvement in mechanical strength. As noted by Wang et al. [49], SAPs application is accompanied by both positive and negative effects on material properties. First, SAPs may serve as an internal curing agent that reduces autogenous shrinkage and porosity. However, this effect dominates in the case of high-performance concrete rather than in low-strength plasters. On the other hand, the left pores from SAPs have an adverse effect on mechanical and flexural strength. The mechanical strength depends on the compatibility of SAPs with the material matrix. In this regard, effects of the chemical composition as well as particle size distribution were described by Ma et al. [35], who linked the swelling and deswelling in a water solution with SAP characteristics. Coarser SAPs having lowered specific surfaces are deemed as less swelling and thus are not accompanied by the empty void formation. Likewise, the reduced deswelling rate creates more stable internal curing conditions, so the desorption rate correlates with the formation of an interfacial transition zone since it affects the development of the annular curing zone. Notwithstanding, this phenomenon is today not clearly understood due to a limited knowledge base. Specifically, not all water released by SAPs is consumed by unhydrated particles and is rather transported by capillary pores. The formed interfacial transient zone (ITZ) around SAPs differs from the ITZ surrounding the regular aggregates, but is much like the ITZ around perlite or other lightweight aggregates. 

### 4.3. Moisture Transport Properties

The water absorption coefficient A, along with the apparent moisture diffusivity κ, is given in Table 5. The water absorption coefficient of plasters containing Creasorb SIS and Cabloc CT increased almost linearly with the increasing SAP dosage; for both PCRE1.5 and PCAB1.5 it was approximately three times higher than for the reference plaster PR. PH mixtures showed a relatively small (~10%) increase of A values up to 1 wt.% of Hydropam addition but PH1.5 had two times higher A than PR. The apparent moisture diffusivity followed the same trends as the water absorption coefficient. The obtained results were in good agreement with the total open porosity (Table 2).

The rapid swelling of SAP particles during PCRE and PCAB inmixing was probably the main factor responsible for the formation of larger voids that can be viewed as a reservoir for moisture uptake. Maturing and drying of the samples then reduced the volume occupied by SAP particles, causing shrinkage of SAP particles. However, a consequent exposure of SAPs to water promoted a reswelling that created small cracks on samples with high SAP dosage, acting as preferential paths for liquid water transport as described by Liu et al. [52]. Nonetheless, this effect was not manifested in PH samples, as the internal stress driven by SAP swelling did not exceed the strength of the material [47]. Our results are contradictory with the findings of Senff et al., who described the adverse effect of SAP admixture on water absorption based on a limited volume of interconnected pores. However, the lack of details about used SAPs reduces the possibility of results comparison. On the contrary, Goncalves et al. [32] enhanced the water absorption by the application of SAPs having a diameter of approx. 100 µm. Arising from the achieved results, the most distinct changes in the water absorption correlate with the particle size distribution of applied SAPs likewise as strength parameters. The increased water sorptivity can be deemed to be a prospective parameter for the design of novel restoration plasters with increased water sorption capability. Compared to frequently used plaster admixtures, achieved results are somewhat similar to the application of perlite [36].

The data obtained for the liquid water transport characteristics in this paper are in good qualitative agreement with the conclusions of researchers who used SAPs as an internal curing agent in concrete mixtures [53,54,55]. It should be noted that both SAP dosages and their characteristics are supposed to be chosen with care to avoid undesired damage during cyclic shrinkage and swelling. The eventual damaging of mortars enhanced by SAPs was discussed by Senff et al. [39], who did not observe any adverse effect of SAPs on material durability. Notwithstanding, this conclusion was based on the application of much lower SAP dosages with a limited impact on functional properties.

The water vapor transport parameters were affected by the application of SAP admixtures to a lower extent than the liquid water transport properties (Table 6) but the trends were very similar. The water vapor diffusion resistance factor μ decreased by up to 50% for PCRE and PCAB plasters and by up to 20% for PH. In this case, the total open pore volume was a more important factor than the volume of the biggest pores, as water vapor transport in smaller pores is much easier than transport of liquid water. The obtained results point to the formation of interconnected pores that enhance water vapor transport properties. The reduction in water vapor resistance factor is not as dramatic as revealed by Goncalves et al. [32]; however, it still provides satisfactory results that can be easily utilized for the moderation of ambient climate. The provided comparison shows more pronounced changes secured by SAPs, when even a relatively small portion has a significant effect on material performance. In this regard, the utilization of fine SAPs brings more significant benefits over coarser SAP particles due to the synergy between the increase in material porosity and the SAP dosage.

### 4.4. Moisture Storage Properties

**Figure 4:**
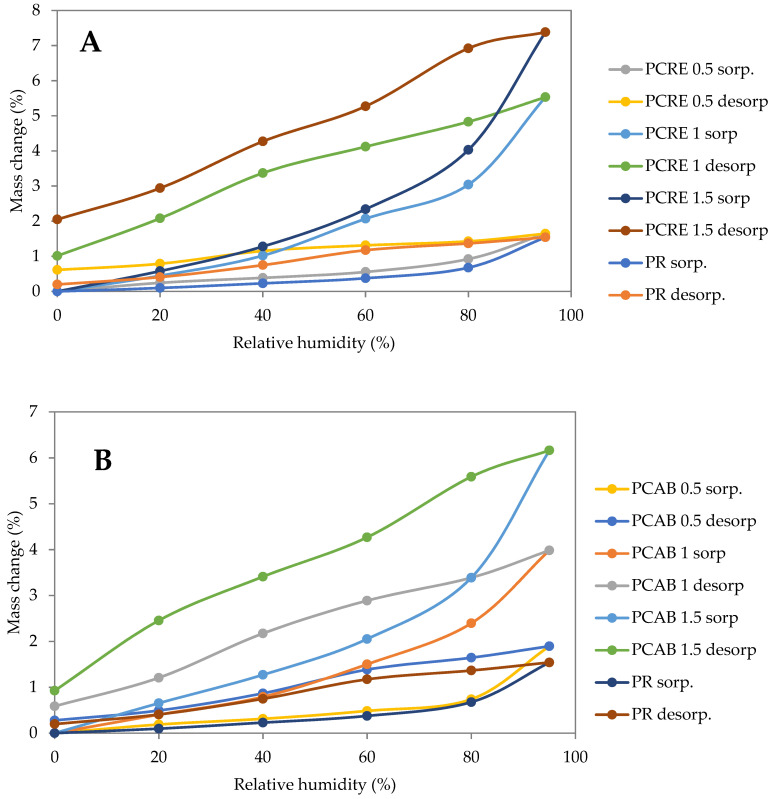

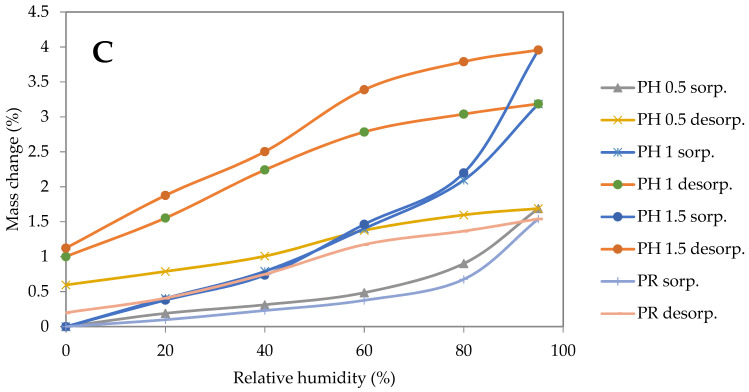
Sorption isotherms of studied plasters: (**A**) PCRE, (**B**) PCAB, (**C**) PH.

Figure 4 shows that 0.5 wt.% dosages of all three analyzed SAPs had very little effect on the water adsorption capability of lime-cement plasters in the hygroscopic range. The most pronounced SAP influence on the amount of adsorbed moisture was observed between 0.5 wt.% and 1 wt.%, where the moisture content of all SAP-modified plasters increased approximately two times over the whole range of relative humidity of 0–95%. The further increase of SAP dosage to 1.5 wt.% brought only an up to 25% increase of moisture adsorbed in the PCRE and PCAB plasters and a negligible change in the hygroscopic behavior of PH. Considering the effects of particular SAPs, the coarser SAP admixture did not affect the moisture storage to such a great extent as for PCRE mixtures. The increasing SAP dosage also led to a substantial increase of hysteresis of sorption isotherms which could be considered as a positive feature from the point of view of the capability of the analyzed plasters to moderate the interior microclimate. A similar phenomenon describing a significantly faster sorption rate compared to desorption rate was also noted by Senff et al. [36]. Considering the initial swelling during the material mixing, the formed voids serve as water/hydrogel reservoirs that can be periodically saturated.

**Figure 5:**
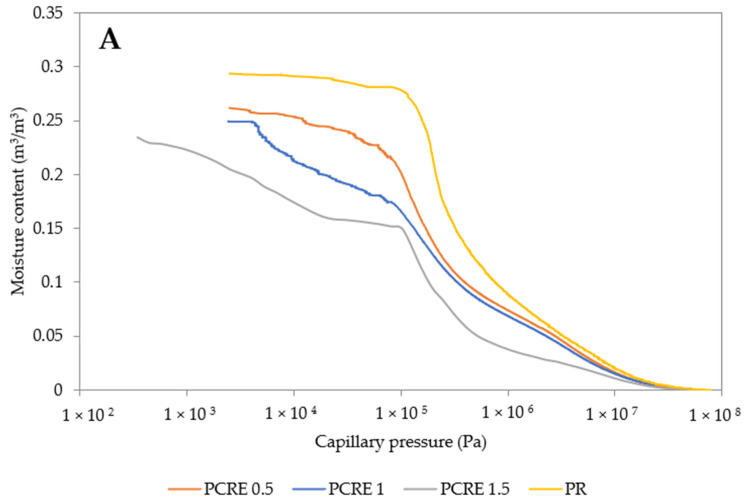

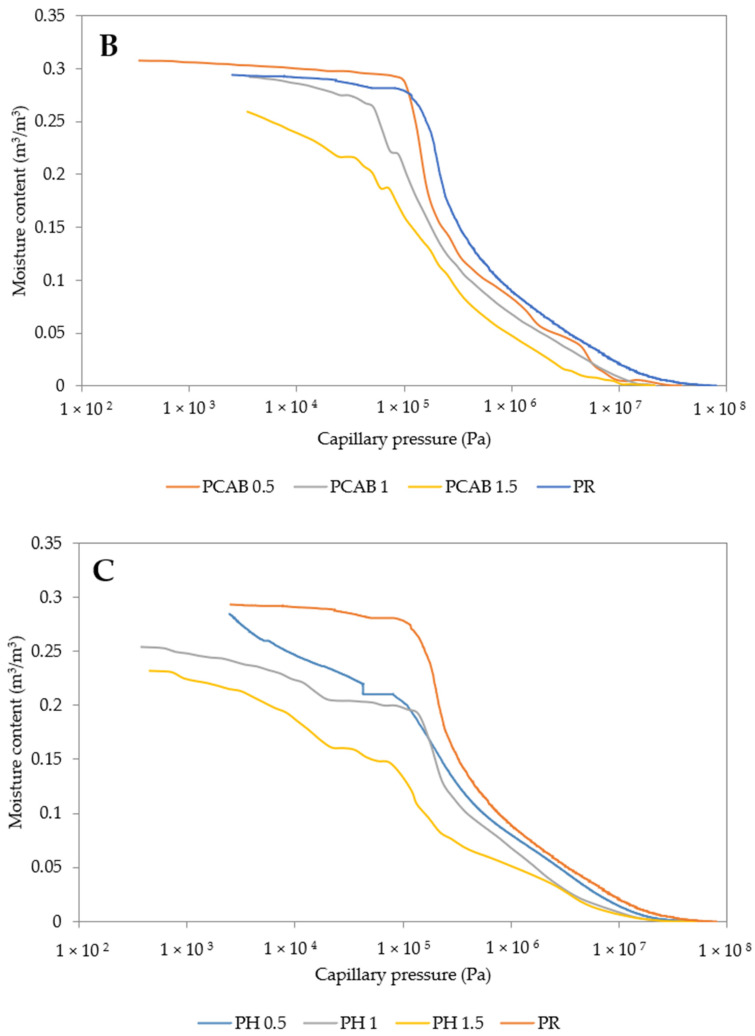
Moisture retention curves of studied plasters: (**A**) PCRE, (**B**) PCAB, (**C**) PH.

The moisture retention curves showed a fast decrease of capillary pressure corresponding to the same moisture content with the increasing SAP dosage (Figure 5). This feature was related to the increased porosity (Figure 3) and could also be considered as positive for interior plasters with a humidity control function. On the other hand, here very minor variation was obtained, thus material performance is better depicted by sorption and desorption curves. As shown in provided figures, most of the desorption curves exhibit a relatively slow rate of water vapor release in ranges between 95% and 80% RH, in particular. On the other hand, the desorption rate accelerates after being dropped below 80% RH.

**Figure 6:**
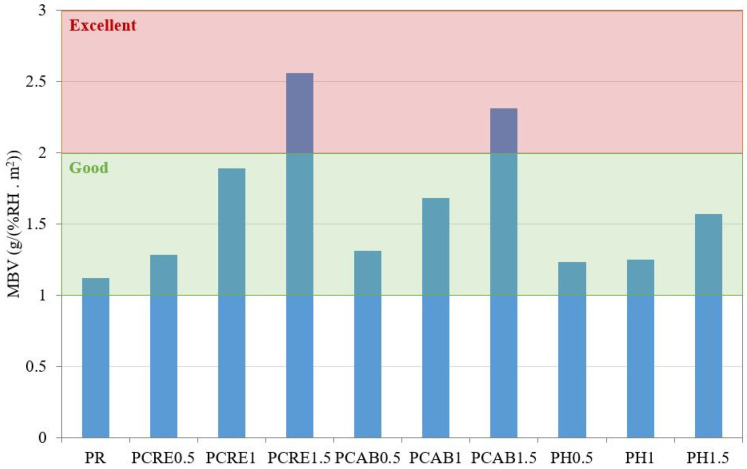
Moisture buffer values of studied plasters.

The moisture buffer values (MBV) determined according to the Nordtest method are presented in Figure 6, together with the classification of particular plasters. The cyclic exposure to different relative humidity levels resulted in substantial differences in the material response of particular plasters. The increased hygroscopicity of applied SAP admixtures led to a continuous increase in sample mass after each cycle. The modified materials revealed thus the capability to compensate for relative humidity fluctuations. While the reference plaster could be classified as good [16], together with most of the other analyzed plaster mixtures, PCRE1.5 and PCAB1.5 showed excellent moisture buffering. The MBV values corresponded well with the sorption isotherms in most cases. The only exception was the PH plaster, where the MBV increase between 1 wt.% and 1.5 wt.% dosages was substantial while sorption isotherms were almost the same. It should be noted that individual classes contain a relatively wide range of moisture performance since PCRE1 represents an improvement of about 70%; however, they still remain in the same category as the reference plaster. 

In a comparison of MBV values measured for SAP-modified plasters in this paper with the results obtained by other investigators for different plasters designed with the same goal, SAPs utilization seems to be very efficient. Jiang et al. [56], who used hemp powder as a hygroscopic agent, achieved only moderate improvement; the studied bio-clay plaster remained classified as good, with MBV ranging between 1.2 and 1.6 g/(m^2^·% RH). MBV obtained by the utilization of perlite, sepiolite, or vermiculite showed only limited improvement <25% [32,38]. Goncalves et al. [32], who applied aluminum powder and sodium olefin-sulfonate for the modification of pore volume, achieved controversial results with SAP-modified materials, as prospective MBV values were accompanied by a substantial worsening of other functional properties.

While both sorption isotherms and MBV describe the moisture storage capacity of building material in the hygroscopic range, their ranges of application are different. 

MBV is a very useful parameter for the building practice in the case where a simple decision between several potential humidity control materials is required [45]. However, for more complex multi-layered building envelopes or those with less common sequencing of layers, its utilization may not be sufficient. For instance, in the walls provided with interior thermal insulation systems without vapor barrier, an assessment of mutual hygric and thermal interactions of particular layers is a necessity and its reduction just to the interior plaster would not lead to a reliable conclusion. Therefore, MBV should be understood as an empirical criterion suitable for a basic assessment of moisture storage capability of humidity control plasters only. 

Sorption isotherm and moisture retention curve are objective physical quantities conforming to the theoretical principles of continuum physics, which can be further used directly in the moisture balance equation [31]. Therefore, such data usually serve as input parameters for computational models of moisture transport [57]. On the other hand, these parameters alone do not make it possible to assess the effects of interior plasters on the interior microclimate directly. Perhaps it would be possible to develop for sorption isotherms a quantitative assessment system similar to MBV but such a system probably would not provide substantially better information. 

High-quality experimental data on the heat and moisture transport and storage properties of interior plasters with supposed humidity control function are indispensable for any serious assessment of their effect on the indoor relative humidity conditions. Nevertheless, the effect of interior plasters on the interior microclimate, although it is definitely a very significant one, may sometimes be overrated, particularly if the properties of the other layers of the envelope are not taken into account. For instance, the water vapor transport parameters of exterior plasters will certainly influence the hygric conditions in the wall very substantially, accentuating or reducing the effect of the exterior environment. Consequently, the relative humidity in the interior will also be affected. The choice of the exterior thermal insulation layer may be similarly important. Therefore, the building envelope should always be considered as a system of layers where not only their hygric and thermal properties but also their sequencing must be taken into account. 

### 4.5. Thermal Properties

**Figure 7:**
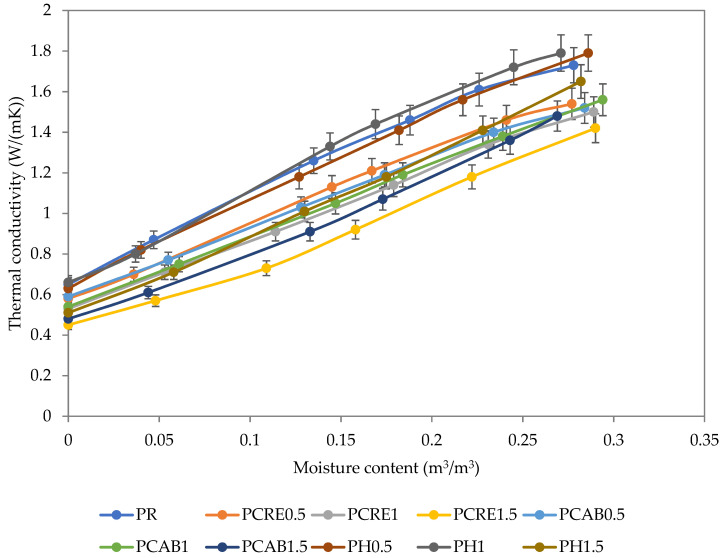
Thermal conductivity of studied plasters.

The thermal conductivity in the dry state of the PCRE and PCAB plasters decreased monotonically, up to 30%, with the increasing SAP dosage (Figure 7). For PH samples, this effect was observed only in the case of PH1.5. The application of Hydropam up to 1 wt.% resulted in only negligible changes of thermal conductivity; for the PH1.5 plaster, the decrease was ~20%. The observed differences comply with the measurements of total open porosity (Table 2). 

The moisture content influenced the thermal conductivity of all analyzed plasters considerably; it raised it up to three times as compared with the dry state, but the trends of differences between the particular plasters observed for the thermal conductivity in the dry state were maintained in the whole range of moisture up to saturation. 

The achieved reduction of thermal conductivity of the analyzed plasters resulting from the SAP application provided an additional benefit to their humidity control function by contributing to the improvement of the overall thermal performance of building envelopes.

**Figure 8:**
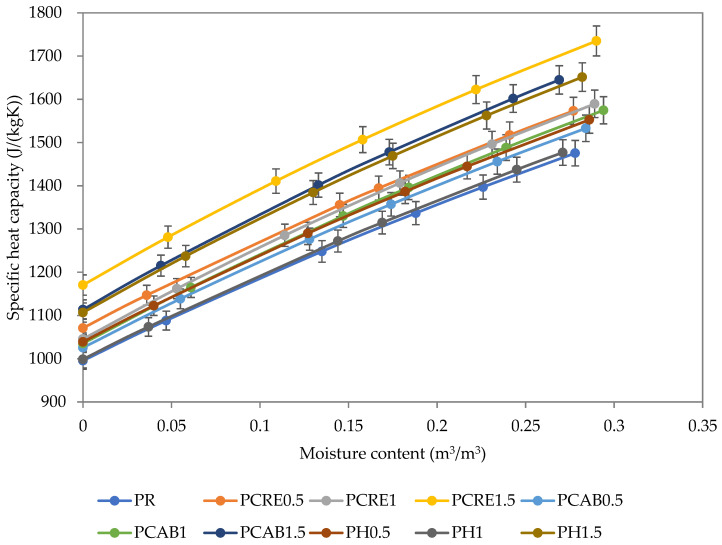
Specific heat capacity of studied plasters.

The specific heat capacity increased with the growing SAP amount in the plaster mixes but the increase was not quite unvarying (Figure 8); this could be attributed to the measurement uncertainty of the applied pulse method. The very significant increase of specific heat capacity of all analyzed plasters with the increasing moisture content was related to the high specific heat capacity of water.

## 5. Conclusions

Several humidity control interior plasters based on the application of three different types of superabsorbent polymers (SAP) were designed, and a wide set of their material properties was analyzed in relation to microstructure. The main results can be summarized as follows:

- The solubility of the applied SAPs in water significantly affected the rheology of fresh mixtures and also consequently the texture of hardened plasters. For insoluble Creasorb SIS and Cabloc CT, an increased pore volume was observed in all pore size intervals while the utilization of soluble Hydropam led up to 1 wt.% dosage even to a certain reduction of pores smaller than 10 μm.
- The results of compressive and flexural strength measurements showed that 1 wt.% SAP dosage could be considered as a threshold value still guaranteeing a satisfactory mechanical performance of the analyzed plasters, despite the differences in chemical composition and solubility in water of the applied SAPs.
- The measurements of water adsorption capability of studied plasters in the hygroscopic range revealed that for all three analyzed SAPs, the most pronounced influence on both the amount of adsorbed moisture and sorption hysteresis was observed when the experimental data for 0.5 wt.% and 1 wt.% were compared. The 1 wt.% SAP dosage thus appeared as the most rational choice, particularly taking into account the corresponding values of mechanical parameters.
- The moisture transport parameters increased with the increasing SAP dosage. For the water absorption coefficient and moisture diffusivity, this effect was more pronounced than for the water vapor diffusion coefficient, which was related to the higher volume of pores larger than 10 μm. These pores acted as preferential paths for liquid water transport. Faster water and water vapor transport is a positive feature for a humidity control plaster as it can enhance the sensitivity of such plasters to the changes in interior hygric conditions.
- The thermal conductivity in the dry state decreased with the increasing SAP dosage, which could be considered as an additional benefit of the SAP used in building envelopes.

The experimental results presented in this paper can serve primarily as a comparison with other material solutions reported by other investigators until now and in the future. In this way, basic information on the suitability of particular plasters as humidity control layers can be obtained and utilized by designers. However, for more complex multi-layered building envelopes or those with less common sequencing of layers, just a comparison of material parameters may not be sufficient. In this case, more sophisticated assessment methods would be required, based on the identification of mutual interactions between the building envelope as a whole and the interior microclimate. The complete sets of basic physical characteristics and hygric and thermal properties of the analyzed plasters determined in this paper can then bring additional benefits. Their added value consists in their direct applicability in computational models of heat and moisture transport as high-quality input data which are indispensable for any advanced assessment of the effect of a particular building envelope on the indoor relative humidity conditions. 

## Figures and Tables

**Table 1:** Absorption of selected SAPs.

	Absorption (g/g Dry SAP)		
	Distilled Water	Tap Water	5 w/c
Creasorb SIS	73	48	22
Cabloc CT	254	128	18
Hydropam	84	52	23

**Table 2:** Composition of studied plaster mixture—reference plaster—PR, plasters with Creasorb SIS—PCRE, Cabloc CT—PCAB, and Hydropam—PH.

Mixture	Dry Plaster Mixture (kg)	w/ds (-)	SAP (g)
PR	1	0.160	-
PCRE0.5	1	0.192	5
PCRE1	1	0.245	10
PCRE1.5	1	0.299	15
PCAB0.5	1	0.205	5
PCAB1	1	0.265	10
PCAB1.5	1	0.335	15
PH0.5	1	0.175	5
PH1	1	0.202	10
PH1.5	1	0.220	15

**Table 3:** Basic material properties of studied plaster mixtures.

Mixture	Bulk Density (kg/m^3^)	Matrix Density (kg/m^3^)	Total Open Porosity (%)
PR	1567	2874	45.5
PCRE0.5	1438	2869	49.9
PCRE1	1377	2778	50.4
PCRE1.5	1256	2803	55.2
PCAB0.5	1482	2857	48.1
PCAB1	1420	2841	50.0
PCAB1.5	1284	2850	54.9
PH0.5	1463	2725	46.3
PH1	1532	2812	45.5
PH1.5	1310	2788	53.0

**Table 4:** Compressive and flexural strength of studied plasters.

Mixture	Compressive Strength (MPa)	Flexural Strength (MPa)
PR	3.87	1.76
PCRE0.5	3.69	1.72
PCRE1	2.98	1.70
PCRE1.5	1.47	1.40
PCAB0.5	3.74	1.96
PCAB1	3.59	1.84
PCAB1.5	2.91	1.45
PH0.5	4.00	2.12
PH1	4.30	2.79
PH1.5	3.23	1.79

**Table 5:** Liquid water transport properties of studied plasters.

Mixture	A (kg·m^−2^·s^−1/2^)	κ (m^2^ s^−^^1^)
PR	0.0798	1.61 × 10^−^^8^
PCRE0.5	0.1369	2.91 × 10^−^^8^
PCRE1	0.1875	2.87 × 10^−^^7^
PCRE1.5	0.2547	5.70 × 10^−^^7^
PCAB0.5	0.1196	2.74 × 10^−^^8^
PCAB1	0.1638	4.68 × 10^−^^7^
PCAB1.5	0.2391	6.23 × 10^−^^7^
PH0.5	0.0955	3.08 × 10^−^^8^
PH1	0.0926	2.76 × 10^−^^8^
PH1.5	0.1463	4.11 × 10^−^^7^

**Table 6:** Water vapor diffusion properties of studied plasters.

Mixture	µ (-)	D (m^2^/s)
PR	10.8	1.56 × 10^−^^6^
PCRE0.5	9.3	1.73 × 10^−^^6^
PCRE1	7.3	2.01 × 10^−^^6^
PCRE1.5	5.4	2.63 × 10^−^^6^
PCAB0.5	9.9	1.69 × 10^−^^6^
PCAB1	7.1	2.05 × 10^−^^6^
PCAB1.5	5.6	2.59 × 10^−^^6^
PH0.5	10.5	1.58 × 10^−^^6^
PH1	10.1	1.58 × 10^−^^6^
PH1.5	8.3	1.82 × 10^−^^6^

## Data Availability

The data presented in this study are available upon request from the corresponding author.
